# Kernel Analysis Based on Dirichlet Processes Mixture Models

**DOI:** 10.3390/e21090857

**Published:** 2019-09-02

**Authors:** Jinkai Tian, Peifeng Yan, Da Huang

**Affiliations:** 1 State Key Laboratory of High Performance Computing, National University of Defense Technology, Changsha 410073, China; 2 College of Computer, National University of Defense Technology, Changsha 410073, China

**Keywords:** Dirichlet processes, Dirichlet processes mixture models, Gaussian mixture models, kernel, spectral analysis

## Abstract

Kernels play a crucial role in Gaussian process regression. Analyzing kernels from their spectral domain has attracted extensive attention in recent years. Gaussian mixture models (GMM) are used to model the spectrum of kernels. However, the number of components in a GMM is fixed. Thus, this model suffers from overfitting or underfitting. In this paper, we try to combine the spectral domain of kernels with nonparametric Bayesian models. Dirichlet processes mixture models are used to resolve this problem by changing the number of components according to the data size. Multiple experiments have been conducted on this model and it shows competitive performance.

## 1. Introduction

Probabilistic models are essential for machine learning to have a correct understanding of problems. While frequentist models are considered a traditional method for statisticians, Bayesian models are more widely used in machine learning fields. A Bayesian model is a statistical model where probability is used to represent all uncertainties in the model. Uncertainty of both input and output are under consideration in a Bayesian model. A parametric Bayesian model assumes a finite set of parameters which is not flexible because the complexity of the model is bounded while the amount of data is unbounded. A Bayesian nonparametric model is a Bayesian model on infinite-dimensional parameter space. The amount of information captured by the model from the data can grow as the amount of the data grows. These models are always connected with some kinds of stochastic processes to describe an infinite number of parameters. For example, the Gaussian process (GP) and Dirichlet process mixture models (DPMM) are two cornerstones of Bayesian nonparametric models.

Dirichlet processes were first introduced in Reference [1]. The stick-breaking methods [2], Pólya urn scheme [3] and the Chinese restaurant process (CRP) [4,5] are several different definitions and constructions of Dirichlet processes. Dirichlet processes are the basis for many Bayesian nonparametric models and are used to process spatial data [6,7], tree-structured data [8,9], relational data [10,11] and sequential data [12,13,14,15].

The recent development of deep learning has led to the extensive use of the neural network in a variety of domains and a higher performance than conventional methods is achieved. However, this is less than satisfactory for statisticians because of the uninterpretability of black-box function approximation. Neal [16] shows that as the number of hidden unions approaches infinity, Bayesian neural networks converge to Gaussian processes. Thus Gaussian processes are regarded as powerful and interpretable alternatives to neural networks. It provides a principled, practical and probabilistic approach to learning in kernel machines [17].

The property of a Gaussian process is closely related to its kernel (or covariance function). Kernels provide a depiction of smoothness and periodicity for models and parameter selection of kernel is essential for model training. There are many widely used kernels, for example, the squared exponential (SE) kernels, rational quadratic kernels, polynomial kernels and the Matérn kernels family. The expressiveness of a kernel is defined as the ability to discover the inherent patterns of the data.

Recent research [18,19,20] has proposed some expressive kernels to discover patterns without human intervention. Deep architectures are used in designing kernels. Other previous work [21,22] sought to combine the Gaussian process into a Bayesian neural network framework. However, these approaches usually do not have closed forms and are thus less interpretable. Meanwhile, sophisticated approximate inference techniques, for example, variational inference and expectation propagation, are required in the training step. Combining and modifying existing kernels to make new kernels is straightforward because the sum, the product or the convolution of two kernels is also a kernel. There are many challenges [23,24] to designing kernels according to this principle for specialized applications. Specific restrictions are enforced on these kernels to avoid overfitting and complicated inference. However, it is hard to generalize these kernels to other applications for their specialized kernel structures.

We use Gaussian mixture models (GMM) to analyze the spectral density of popular kernels and find that traditional GMM suffer from overfitting and underfitting by a fixed number of components. Therefore, we seek to combine an infinite mixture model in designing kernels. This innovation introduces more flexibility and expressiveness into designed kernels.

Our work is distinct in that we combine Dirichlet process mixture models into analyzing and designing kernels from the spectral domain. Using traditional Gaussian mixture models in kernel analysis [25] imposes restraint on the expressiveness of kernels. Dirichlet process mixture models are flexible models to fit data because the complexity of models grows as more data are observed. By using it to our advantage, we propose the infinite mixture (IM) kernel. The combination of these two traditional Bayesian nonparametric models is one of the most innovative contributions of this work. Theoretically, we prove that with possibly infinite components in mixture models, designed kernels can fit any linear or nonlinear function by arbitrary accuracy. Besides, a Markov Chain Monte Carlo (MCMC) implementation of a hierarchical Bayesian model for spectral analysis is presented and experiments are conducted on real-world temporal data which show the IM kernel’s competitiveness in comparison with other kernels and its superiority in extrapolation.

To the best of our knowledge, this is the first trial to combine the Dirichlet process mixture model in designing kernels. The result is promising on account of the computational convenience and expressiveness of Dirichlet process mixture models. Our infinite version has several advantages. (1) We use MCMC methods (collapsed Gibbs sampling specifically) to avoid the local minimum problem which frequently occurs in derivative-based methods like EM used in conventional GMM. (2) The number of Gaussian components *K* is automatically determined by the provided data, which alleviates the requirement for expertise. On the other hand, it is beneficial for the customization of a kernel for a specific scene. (3) Setting *K* in advance limits the expressiveness of kernels, while an infinite version makes more details available.

The main contribution of the paper can be summarized in two parts:

- We try to combine a hierarchical Bayesian model with the spectral mixture kernel. Our method shows better and more robust performance.
- The original spectral mixture kernel initializes the mean of each component uniformly between 0 and the Nyquist frequency. We analyze the robustness of the spectral mixture kernel and find this strategy to have fluctuant performance. One of the strongest advantages of our method is that the infinite version has a better solution for initialization and has more stable performance.

In Section 2, we have a brief review of the Dirichlet process and the Gaussian process. We introduce a hierarchical Dirichlet mixture model in Section 3 and use this model to analyze kernels in the spectral domain and put forward the concept of *the infinite mixture kernel* in Section 4. In Section 5, we conduct multiple experiments to show the robustness and good performance of the IM kernel.

## 2. Background

### 2.1. Dirichlet Distribution

Dirichlet distribution is an extension of Beta distribution from bivariate to multivariate and can be expressed as:

(1) $$f\left(x_{1},\ldots ,x_{K};\alpha_{1},\ldots ,\alpha_{K}\right)=\frac{1}{B(\alpha )}\prod_{i=1}^{K}x_{i}^{\alpha_{i}-1},$$

where $x_{1},\ldots ,x_{K}>0$ and $\sum_{i=1}^{K}=1$. The Beta function B(α) is the normalizer of the distribution and can be expressed in the form of Gamma function:

(2) $$B\left(\alpha \right)=\frac{\prod_{i=1}^{K}\Gamma \left(\alpha_{i}\right)}{\Gamma \left(\sum_{i=1}^{K}\alpha_{i}\right)},\;\alpha =\left(\alpha_{1},\ldots ,\alpha_{K}\right).$$

Thus we can regard a sample from the Dirichlet distribution $\left(x_{1},\ldots ,x_{K}\right)$ as a distribution itself.

The probability density function (PDF) of the gamma distribution is:

(3) $$p\left(x|\alpha ,\theta \right)=G\left(\alpha ,\theta \right)=\frac{x^{\alpha -1}e^{-x/\theta }}{\Gamma \left(\alpha \right)\theta^{\alpha }}.$$

There is another version of the PDF of the gamma distribution which is also widely used:

(4) $$p\left(x|\alpha ,\theta \right)=G_{R}\left(\alpha ,\theta \right)=\frac{x^{\alpha /2-1}e^{-\alpha x/2\theta }}{\Gamma \left(\alpha /2\right){\left(2\theta /\alpha \right)}^{\alpha /2}}.$$

The transformation between these two versions is:

(5) $$\begin{array}{cc} G(\alpha ,\theta ) & =G_{R}\left(2\alpha ,\alpha \theta \right) \end{array}$$

(6) $$\begin{array}{cc} G_{R}\left(\alpha ,\theta \right) & =G(\frac{\alpha }{2},\frac{2\theta }{\alpha }). \end{array}$$

### 2.2. Dirichlet Process

Dirichlet processes (DP) are a family of stochastic processes whose observations are probability distributions. A Dirichlet process has two parameters, a base distribution *H* and a positive real-valued scalar α called scaling or concentration parameter. Given these two parameters and a measurable set *S*, the Dirichlet process DP(H,α) is a stochastic process whose sample path is a probability distribution over *S*, such that the following holds:

For any finite partition of *S*, denoted $B_{i=1}^{n}$, if

(7) G∼DP(H,α),

then

(8) $$(\left(G\left(B_{1}\right),\ldots ,G\left(B_{n}\right)\right)∼\mathrm{Dir}\left(\alpha H\left(B_{1}\right),\ldots ,\alpha H\left(B_{n}\right)\right).$$

The concentration parameter α is considered the pseudo count number and the base distribution *H* can be considered the prior knowledge of the distribution [26].

### 2.3. Gaussian Process

The Gaussian process is widely used as a statistical model for modeling functions. In the field of machine learning, we are not especially interested in drawing random function from the prior. There always exist raw training data or evaluation results of a model from a series of experiments iterations. These data provide extra knowledge of the model and incorporating this knowledge into the model is of primary interest. In the Gaussian process regression, we seek to predict f(x) based on limited observations.

We express Gaussian process as:

(9) $$f\left(x\right)∼\mathrm{GP}(m\left(x\right),k\left(x,x^{\prime }\right)).$$

Suppose f(x) is a real process, we can define the mean function m(x) and the covariance function $k(x,x^{\prime })$ as:

(10) $$\begin{array}{cc} m(x) & =E[f(x)],\\ k(x,x^{\prime }) & =E[\left(f\left(x\right)-m\left(x\right)\right)\left(f\left(x^{\prime }\right)-m\left(x^{\prime }\right)\right)]. \end{array}$$

## 3. A Hierarchical Dirichlet Process Mixture Model

A Gaussian mixture model can be written as:

(11) $$p\left(y|\mu_{1},\ldots ,\mu_{K},\Lambda_{1},\ldots ,\Lambda_{K},\pi_{1},\ldots ,\pi_{K}\right)=\sum_{i=1}^{K}\pi_{i}N\left(\mu_{i},\Lambda_{i}^{-1}\right),$$

where $\mu_{i}$ denotes the mean of the *i*-th component, $\Lambda_{i}$ denotes the precision of the *i*-th component and $\pi_{i}$ denotes the weight or mixture coefficient of the *i*-th component which satisfies $\sum_{i=1}^{K}\pi_{i}=1$ and $\pi_{i}\geq 0$ for i=1,…,K. A GMM model can be learned using expectation–maximization (EM) algorithm. However, the number of components *K* is set manually here which is inconvenient in practical applications because *K* should vary with the complexity of data.

Given hyperparameters for means, precisions and weights respectively, a hierarchical model in high dimension with a potentially infinite number of Gaussian components is implemented. We first consider inference in a finite version with *K* components and then extend it into infinite hierarchical models as the limiting case of the finite one.

**Figure 1:**
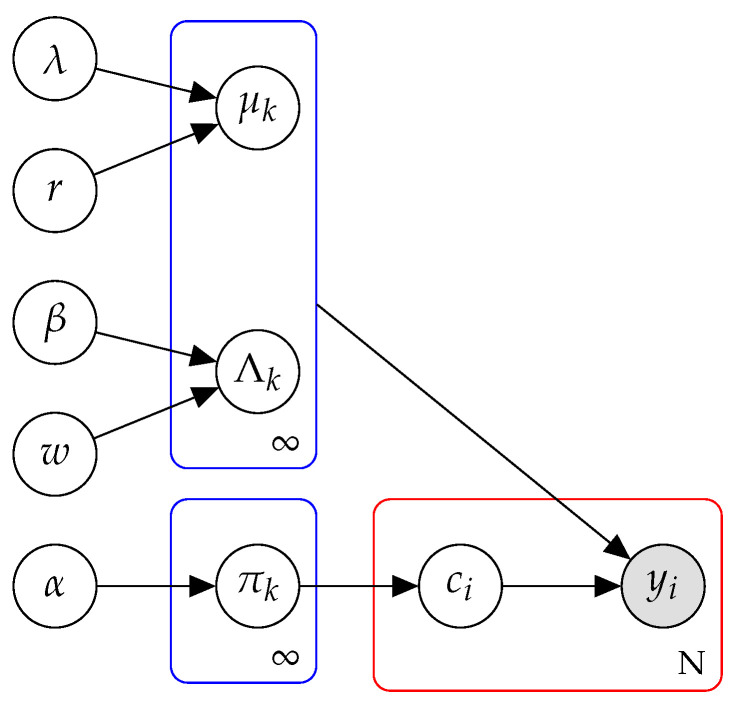
Graphical model representation of an infinite hierarchical mixture model. Nodes denote random variables. Under the condition of high dimensionality, each node can be a collection of multiple random variables. Edges denote dependence. Two blue plates denote replication of global hidden variables and the red plate denotes replication of local hidden variables and observations. Notice that blue plates have potentially infinite number of replications, while the red plate has only *N* replications corresponding to each data point.

We outline our model in a probabilistic graphical model for clarity. Figure 1 illustrates the hierarchical structure embedded in the model. Nine nodes represent different collections of random variables and one node represents observations. We classify these random variables into hyperparameters, global variables and local variables. The first column contains hyperparameters with priors set by sufficient statistics from the data. The second column contains means, precisions and weights of *K* components which we call global variables. The node tagged by $c_{i}$ represents the local variable that indicates which component generates this data point.

In Gaussian mixture models, the data ${y_{j}}_{j=1}^{N}$ is assumed to come from a generative model:

(12) $$y_{j}|c_{j}∼N\left(\mu_{c_{j}},\Lambda_{c_{j}}^{-1}\right).$$

To establish a hierarchical model, we put priors on $\mu_{i}$ and $\Lambda_{i}$:

(13) $$p\left(\mu_{i}|\lambda ,r\right)∼N\left(\lambda ,r^{-1}\right),\;p\left(\Lambda_{i}|\beta ,w\right)∼G\left(\beta ,w^{-1}\right),$$

where G denotes the gamma distribution. First we have a deeper insight into μ. We give hyperparameters λ and *r* vague priors as:

(14) $$p\left(\lambda \right)∼N\left(\mu_{y},\Sigma_{y}\right),$$

(15) $$p\left(r\right)∼G\left(\frac{1}{2},2\Sigma_{y}^{-1}\right)\propto r^{-1/2}\exp\left(\frac{-r\Sigma_{y}}{2}\right),$$

where $\mu_{y}=\frac{1}{N}\sum_{j=1}^{N}y_{j}$ and $\Sigma_{y}=\frac{1}{N}\sum_{j=1}^{N}{\left(y_{j}-\mu_{y}\right)}^{2}$ denote the mean and variance of all observations.

When considering prior in Bayesian inference, the usage of conjugate priors allows the results to be derived in closed form. It is conventional to use Gaussian, gamma and inverse-gamma distribution as conjugate priors for scalar means, precisions and variances, respectively [27]. Considering the Wishart distribution is a generalization of the gamma distribution to multiple dimensions, we use Gaussian, Wishart and inverse-Wishart distribution as conjugate priors for high-dimensional hyperparameters. The posterior distribution of μ given other variables is:

(16) $$p\left(\mu_{i}|\lambda ,r,\Lambda_{i},c,y\right)∼N\left(\lambda +\frac{\left({\bar{y}}_{i}-\lambda \right)}{n_{i}\Lambda_{i}+r}n_{i}\Lambda_{i},{\left(n_{i}\Lambda_{i}+r\right)}^{-1}\right),$$

where $n_{i}=\sum_{j=1}^{N}\delta \left(c_{j},i\right)$ denotes the number of data points generated by the *i*-th component and ${\bar{y}}_{i}=\frac{1}{n_{i}}\sum_{j:c_{j}=i}y_{j}$ denotes the mean of observations in this component. As to hyperparameters, taking Equation (14) and Equation (15) as priors and Equation (13) as likelihood, we get the posteriors:

(17) $$\begin{array}{cc} p(\lambda |\mu_{1},\ldots ,\mu_{K},r) & ∼N\left(\mu_{y}+\frac{\sum_{i=1}^{K}\mu_{i}-K\mu_{y}}{\Sigma_{y}^{-1}+Kr}r,{\left(\Sigma_{y}^{-1}+Kr\right)}^{-1}\right),\\ p\left(r|\mu_{1},\ldots ,\mu_{K},\lambda \right) & ∼W\left(\frac{1}{D}{\left(\Sigma_{y}+\sum_{i=1}^{K}{\left(\mu_{i}-\lambda \right)}^{⊤}\left(\mu_{i}-\lambda \right)\right)}^{-1},\frac{K+1}{D}\right), \end{array}$$

where W denotes the Wishart distribution and *D* denotes the number of dimensions. The posterior of precision and its hyperparameters can be derived in the same way.

The weight of components $\pi_{1},\ldots ,\pi_{K}$ have a hyperparameter α. We can simply set the prior of α as $p\left(\alpha^{-1}\right)∼G\left(\frac{1}{2},2\right)$, which means $p\left(\alpha \right)\propto \alpha^{-3/2}\exp\left(-\frac{1}{2\alpha }\right)$. The joint distribution of *N* category variables $c_{j}$ is:

(18) $$p\left(c_{1},\ldots ,c_{N}|\pi_{1},\ldots ,\pi_{K}\right)=\prod_{i=1}^{K}\pi_{i}^{n_{i}}.$$

Notice that this distribution is a multinomial distribution with permutation rather than combination and the Dirichlet distribution is the conjugate prior of the multinomial distribution in Bayesian statistics. In order to bring as less prior information as possible, we give a symmetric Dirichlet distribution prior for $\pi_{i}$:

(19) $$p\left(\pi_{1},\ldots ,\pi_{K}|\alpha \right)∼\mathrm{Dirichlet}\left(\alpha /K,\ldots ,\alpha /K\right)=\frac{\Gamma (\alpha )}{\Gamma {\left(\alpha /K\right)}^{K}}\prod_{i=1}^{K}\pi_{i}^{\alpha /K-1}.$$

By combining Equations (18) and (19) we can integrate out the weight:

(20) $$p\left(c_{1},\ldots ,c_{N}|\alpha \right)=\frac{\Gamma (\alpha )}{\Gamma (n+\alpha )}\prod_{i=1}^{K}\frac{\Gamma \left(\alpha /K+n_{i}\right)}{\Gamma (\alpha /K)}.$$

Collapsed Gibbs sampling requires updating one variable each time, so $c_{j}$ for all j=1,…,N should be considered separately. We can derive from Equation (20) directly:

(21) $$p\left(c_{j}=i|c_{-j},\alpha \right)=\frac{n_{-j,i}+\alpha /K}{n-1+\alpha },$$

where $c_{-j}$ denotes $c_{j}$ for i=1,…,j-1,j+1,…,N and $n_{-j,i}$ denotes the number of data points generated by the *i*-th component without the *j*-th data point.

When *K* rounds towards infinity, Equation (21) become $p\left(c_{j}=i|c_{-j},\alpha \right)=n_{-j,i}/\left(n-1+\alpha \right)$. Notice that the probability of the *j*-th data point in all existing components is $\sum_{i=1}^{K}p\left(c_{j}=i|c_{-j},\alpha \right)=\left(n-1\right)/\left(n-1+\alpha \right)$, which is not equal to 1. Thus the probability of this data point belonging to a new component is $p\left(c_{j}\neq c_{j^{\prime }}\;\mathrm{for}\;\mathrm{all}\;j^{\prime }\neq j|c_{-j},\alpha \right)=\alpha /\left(n-1+\alpha \right)$. The mean and precision of the new component are sampled from the prior. In the meantime, components are removed when they get empty. After enough iterations, this model tends to be stabilized. The detailed derivation of all formulas in this section can be found in Appendix A.

## 4. Kernel Analysis in the Spectral Domain

In this section, we analyze kernels in their spectral domain and propose a new kernel called the infinite mixture (IM) kernel. The IM kernel is defined as a kernel with infinite Gaussian mixture components in its spectral domain. Considering the symmetric property of spectral density for stationary kernels, the spectral density of the infinite mixture kernel is designed as:

(22) $$\begin{array}{cc} \; & \theta \left(s_{i}\right)∼N\left(\mu_{i},\Lambda_{i}^{-1}\right),\\ \; & S\left(s\right)=\sum_{i=1}^{\infty }\frac{1}{2}w_{i}\left(\theta \left(s_{i}\right)+\theta \left(-s_{i}\right)\right), \end{array}$$

where $\mu_{i}$, $\Lambda_{i}$ and $w_{i}$ are the mean, precision and weight for the *i*-th component respectively.

The design of a kernel is of essential importance in Gaussian process. When considering interpolation, we need the covariance function to be smooth enough, which means if two input points are close to each other, we expect the value of the function at those points to be similar. This requires the covariance matrix to have the attribute that if two points are closer, the corresponding element in the matrix will be larger. The squared exponential (SE) kernel, also called the radial basis function kernel, is the most widely used kernel and has the form:

(23) $$k\left(x,x^{\prime }\right)=\exp\left(-\frac{{∥x-x^{\prime }∥}^{2}}{2\ell^{2}}\right).$$

It is infinitely differentiable, which means the Gaussian process fitted with this kernel has mean square derivatives in any order and is thus very smooth. A stationary kernel is a function of τ=x-x′. According to the definition, the SE kernel is a typical stationary kernel and can be reformulated as $k\left(\tau \right)=\exp\left(-{∥\tau ∥}^{2}/2\ell^{2}\right)$. The application of the Bochner’s theorem (Theorem 1) [28,29] enables us to analyze a stationary kernel from its spectral domain.

**Theorem** **1.** *A complex-valued function k on $R^{D}$ is the function of a weakly stationary mean square continuous complex-valued random process on $R^{D}$ if and only if it can be represented as:*

(24) $$k\left(\tau \right)=\int_{R^{D}}e^{2\pi is\cdot \tau }d\mu \left(s\right),$$

*where μ is a positive finite measure.*

If μ has a density S(s), that is, dμ(s)=S(s)ds, then *S* is called the spectral density or power spectral of kernel *k*. Furthermore, when the spectral density exists, the Wiener-Khintchine theorem shows that the covariance function and the spectral density are Fourier duals of each other:

(25) $$\begin{array}{c} k\left(\tau \right)=\int S\left(s\right)e^{2\pi is^{⊤}\tau }ds,\;S\left(s\right)=\int k\left(\tau \right)e^{-2\pi is^{⊤}\tau }d\tau . \end{array}$$

By substituting the SE kernel in Equation (23) into (25), we can get the corresponding spectral density of the SE kernel as a Gaussian function $S\left(s\right)={\left(2\pi \ell^{2}\right)}^{D/2}\exp\left(-2\pi^{2}\ell^{2}s^{⊤}s\right)$, where *D* denotes the dimensionality of s. Note that the variance of process is k(0)=∫S(s)ds, which is exactly the integral in spectral domain.

Analyzing kernel from a spectral (or frequency) perspective, the characteristic of zero-centered Gaussian in the spectral density of the SE kernel restricts its expressiveness. Wilson and Adams [25] proposed the spectral mixture (SM) kernel as an extension of traditional kernels by using several Gaussian components instead of one zero-centered Gaussian.

The number of components of the SM kernel is set to a fixed value. However, without additional expertise, setting *K* in advance is not suitable for practical application which gives rise to underfitting or overfitting. When the inherent pattern of data is simple, setting *K* with a large value will take noise in frequency domain into consideration and result in overfitting. Meanwhile, when the inherent pattern is sophisticated, setting *K* with a small value will lead to the ignorance of details and result in underfitting.

In order to address this problem, we combine an infinite mixture model into kernel design and propose the infinite mixture kernel under a Bayesian framework. By combining Equations (25) and (22), we get the analytic expression of the IM kernel:

(26) $$k\left(\tau \right)=\sum_{i=1}^{\infty }w_{i}\exp\left(-2\pi^{2}\tau^{2}\Lambda_{i}^{-1}\right)\cos\left(2\pi \tau \mu_{i}\right),$$

where $\sum_{i=1}^{\infty }w_{i}=1$ and $w_{i}\geq 0$.

The IM kernel can assign *K* automatically as the distribution of data changes. The Wiener’s Tauberian theorem (Theorem 2) gives the theory ground to the assertion that an infinite mixture of Gaussians is dense in the set of all possible distributions.

**Theorem** **2.** *Let $f\in L_{1}\left(R\right)$ be an integrable function. The span of translations $f_{a}\left(x\right)=f\left(x+a\right)$ is dense in $L_{1}\left(R\right)$ if and only if the Fourier transform of f has no real zeros.*

This theorem is stronger because it only uses translation transformation to make the span dense in $L_{1}\left(R\right)$ function space. Various popular kernels can be reconstructed by the IM kernel via analyzing their spectral density which shows the expressiveness of the IM kernels from another point of view.

Before applying infinite mixture models, we need to get the empirical spectral density of the data first. From Theorem 1, the spectral density is proportional to the discrete Fourier transform (DFT) of the squared data. The time complexity of DFT is $O(n^{2})$, which can be reduced to O(nlogn) using fast Fourier transform (FFT). FFT is used here to calculate the spectral density. Notice that if the observations are not equidistant, data should be preprocessed. A simple way to preprocess the data is using interpolation methods like linear interpolation, polynomial interpolation and spline interpolation. A more precise and complex approach is using a compressed sensing algorithm [30]. Further experiments show that when the input has a grid structure except for a lack of input in a few ranges, it works well without preprocessing. We refer to it as a quasi-grid structure. The empirical spectral density is taken as an unnormalized distribution and we get observations by slice sampling [31].

## 5. Experiments

In this section, we conduct a series of experiments to verify the robustness and good performance of the IM kernel on multiple datasets. We first introduce details of the implementation of the hierarchical mixture model and analyze the independence of samples. Then we perform robustness test on the SE kernel and the IM kernel, finding that performance of the SE kernel fluctuates wildly with different random seeds while IM kernel maintains good performance. Finally, we compare IM kernel with other widely used kernels in several datasets. The IM kernel shows smaller negative log-likelihood and mean square error (MSE) than others. Matlab (version 9.1) code to perform all experiments is available on GitHub (https://github.com/deeperKernelInsight/infinitemixturekernel). All experiments are conducted on a PC with a 3.20 GHz Hexa-core CPU and 16 GB RAM.

### 5.1. An MCMC Implementation of the Hierarchical Model

A collapsed Gibbs sampler marginalizes over a part of dependent variables while sampling from other variables. This strategy is widely used in hierarchical Bayesian models like latent Dirichlet allocation (LDA). Collapsing out the Dirichlet distribution in this model helps to accelerate sample steps. We use collapsed Gibbs sampling as the specific MCMC method to sample hyperparameters, local variables and global variables. Meanwhile, the exact order of sampling parameters influences the performance. In the implementation, we sample means and precisions for all components first, with hyperparameters next and indicator variables at last.

**Figure 2:**
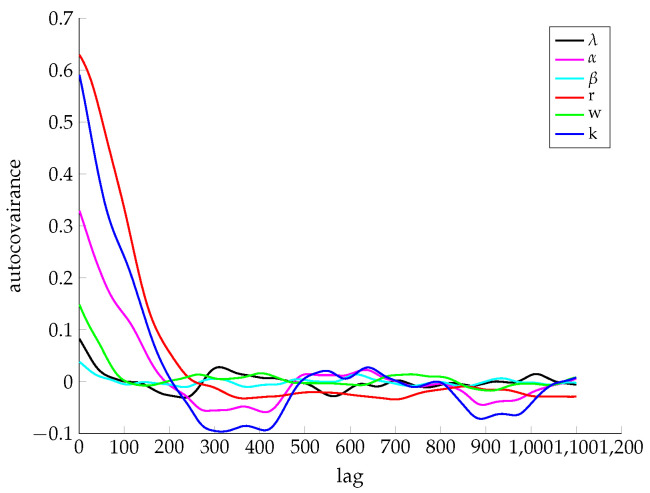
Autocorrelation analysis for hyperparameters.

We use the airline passenger dataset which records monthly passenger numbers from 1949 to 1961 here to look into this MCMC process. Before the Markov chain becomes stationary, there are a few iterations for “burn-in”. In all experiments below, we set 1000 iterations for “burn-in”. Since collapsed Gibbs sampling draws only one parameter each time, observations close to each other tend to be correlated. Figure 2 plots the autocorrelation function of hyperparameters taking lag as *x*-axis. From this figure, the parameters are deemed independent of itself after 200 iterations. After the “burn-in” iterations, we record observations every 100 iterations and choose the most suitable one to refine.

**Figure 3:**
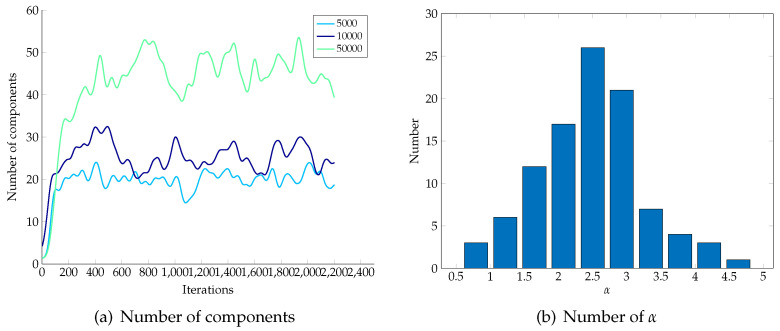
Parameters observed in the process of Markov Chain Monte Carlo (MCMC).

Figure 3a shows the number of components in the process of training with a different number of sampling points. For universality, we set only one component in the beginning. It increases rapidly in the first few iterations and stays stable with slight fluctuations. With the number of sampling points increases, the number of components used to depict the model grows. The primary cause is that models become complex with the growth of sampling points and this is where the flexibility emerges. Figure 3b plots histogram of 100 observations for α in the condition of 10,000 sampling points. The concentrated parameter α takes values around 2.5, which means only α/(n+α)≈0.025% of data points belonging to a new component.

### 5.2. Robustness Test

**Figure 4:**
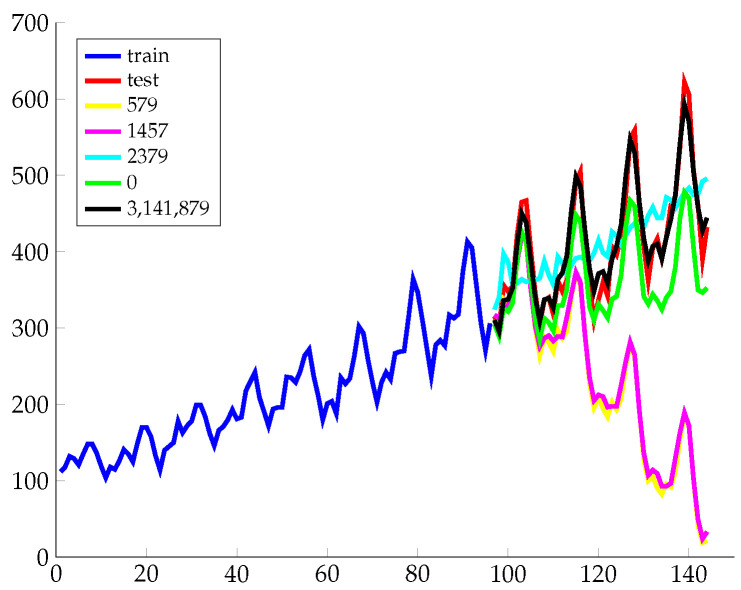
Performance of the spectral mixture (SM) kernel with different random seeds.

Since the learning surface for a spectral mixture kernel is often high multimodal, the original model of the SE kernel is vulnerable for its stern demand for initialization. The SE kernel takes a rather simple and greedy strategy by setting initial weights as k(0)/K, initial variances sampled from a truncated Gaussian distribution with a mean equal to the range of the data and initial frequencies sampled from a uniform distribution from 0 to its Nyquist frequency. Figure 4 shows the performance of the SM kernel with different random seeds. It performs well when the random seed takes the value 3,141,879. However, taking 579 and 1457 as random seeds results in bad performance. Notice that there are obvious performance gaps between random seeds which indicates that the simple strategy used by the SM kernel is not stable and is thus not a good option. Performances of the IM kernel with different random seeds are not plotted because results remain stable and overlap in the chart. By switching the number of sampling points, the performances are slightly different. With a small number of sampling points, kernel tends to show secular trends at the expense of details. Meanwhile, with a large number of sampling points, kernel tends to show more details while secular trend flattens out.

### 5.3. Performance on Multiple Datasets

The datasets we employ are publicly available and has been used in previous research [24]: (a) monthly gasoline demand in Ontario; (b) monthly death tolls from bronchitis in the UK; (c) mean monthly air temperature; (d)weekday bus ridership in Iowa city. We divide these datasets each into two parts with 2/3 data as the training set and others as the testing set. We attempt to give fair treatment to these kernels by initializing hyperparameters with high marginal log-likelihoods and fully training kernels to convergence. The marginal log-likelihood with noise observations can be expressed as:

(27) $$\log p\left(y|X,\theta \right)=-\frac{1}{2}y^{⊤}{\left(K_{\theta }+\sigma_{n}^{2}I\right)}^{-1}y-\frac{1}{2}\log\left|K_{\theta }+\sigma_{n}^{2}I\right|-\frac{n}{2}\log2\pi .$$

We use GPML toolbox (http://www.gaussianprocess.org) to refine parameters and evaluate other popular kernels.

**Figure 5:**
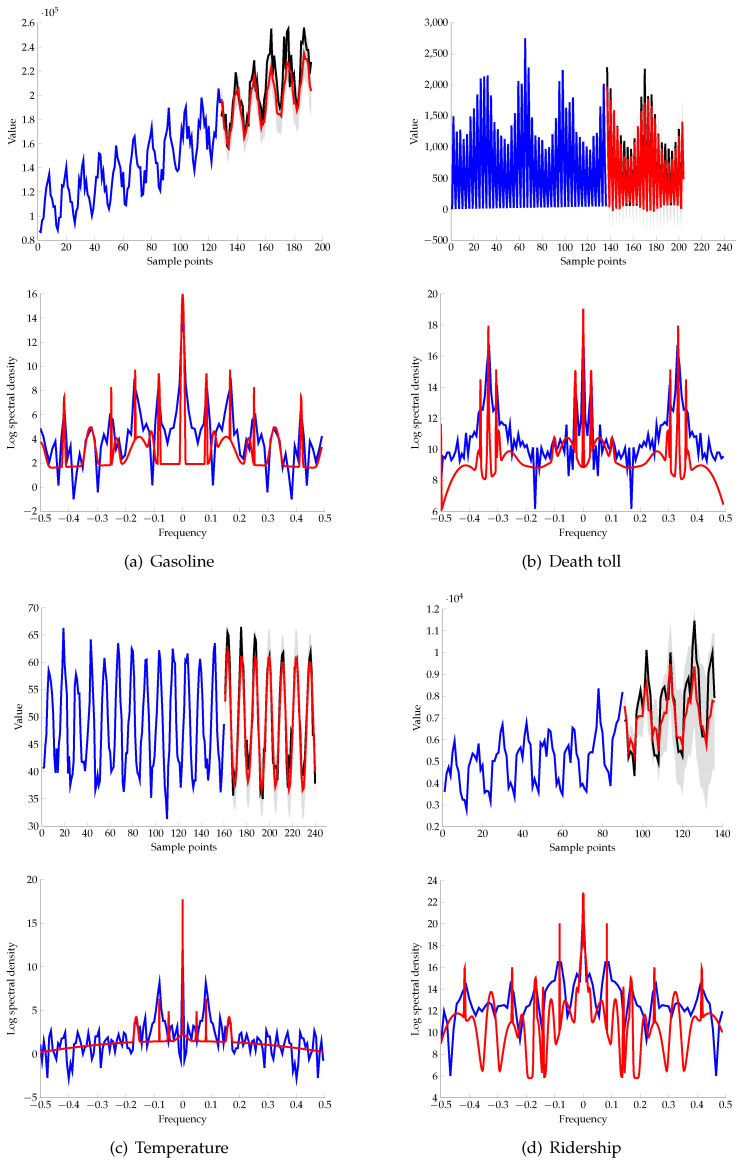
Performance of the infinite mixture (IM) kernel on a bunch of datasets.

Figure 5 shows the predictive distribution of the IM kernel and its spectral density. The top row displays predicting results on the testing set with the training and testing set in blue and black respectively. The mean of the predictive distribution is in red. The IM kernel shows good predictive ability with the testing set located in 95% of the predictive mass shown in shadow. The bottom row shows the log spectral density of the IM kernel in red and the log empirical spectral density in blue. The learned kernel fitted well with the empirical spectral. The log spectral density of kernel in the Temperature dataset shows the ability to filter noise.

Table 1 and Table 2 show negative log-likelihood and MSE of the SE kernel, the Matérn kernel, the squared exponential (SE) kernel and the IM kernel with different numbers of sampling points. In experiments, we set numbers of sampling points as 5000, 10,000 and 50,000. Only the standard deviations of the IM and SM kernels are recorded in the brackets due to the space limitation. The IM kernel shows better performance than others both in negative log-likelihood and in MSE. Meanwhile, when the number of sampling performance increases, the result turns out better. While a larger number of sampling points needs a longer time to sample and inference, the trade-off between accuracy and time cost is concerned in practical application.

## 6. Conclusions

We introduced the infinite mixture kernel by combining a hierarchical Bayesian model into the analyze of the spectral domain. The extensive experiments have shown the robustness and better performance of our method than the finite version. The results are competitive with popularly used kernels and the spectral density of the IM kernel provides an extra interpretation of the inherent pattern of the data.

In future work, one can combine the IM kernel with approximate inference algorithms. What’s more, in this paper we only analyze the kernel of a specific stochastic process. Although the Gaussian distribution and the Gaussian processes are widely used in many different fields, there are many other distributions and their corresponding stochastic processes, for example, the q-Gaussian distribution and the distributions which emerge in the anomalous diffusion contexts and their corresponding stochastic processes. Analyzing the performance of these unconventional distributions and their corresponding stochastic processes can result in interesting discoveries. We believe that kernel analysis from a spectral perspective helps to design more powerful kernels while a deeper investigation is still needed for future research.

## Figures and Tables

**Table 1:** Negative Log-Likelihood. (The results with the best performance are shown in bold).

Dataset	Unit	IM			SM	Matérn	SE
		$5\times 10^{3}$	$1\times 10^{4}$	$5\times 10^{4}$			
Gasoline	$1\times 10^{3}$	1.31(0.09)	1.31(0.07)	**1.27(0.06)**	14.3(0.27)	1.49	1.44
Death toll	$1\times 10^{2}$	8.55(0.14)	8.49(0.20)	**8.48(0.10)**	9.66(0.43)	28,500,000	11.2
Temperature	$1\times 10^{2}$	3.80(0.12)	3.70(0.15)	3.71(0.15)	**3.68(0.29)**	5.71	4.74
Ridership	$1\times 10^{2}$	6.57(0.23)	6.96(0.26)	**6.48(0.14)**	7.01(0.33)	44,100,000	7.66

**Table 2:** Mean Square Error (MSE). (The results with the best performance are shown in bold).

Dataset	Unit	IM			SM	Matérn	SE
		$5\times 10^{3}$	$1\times 10^{4}$	$5\times 10^{4}$			
Gasoline	$1\times 10^{8}$	3.50(0.21)	3.27(0.19)	**2.94(0.13)**	6.18(0.76)	335,000,000	394
Death toll	$1\times 10^{4}$	1.74(0.06)	**1.67(0.03)**	1.83(0.03)	6.35(0.16)	1,040,000	80.7
Temperature	1	7.05(0.10)	6.75(0.32)	**5.89(0.10)**	6.85(0.69)	81.1	1850
Ridership	$1\times 10^{6}$	2.32(0.08)	3.12(0.11)	**1.14(0.13)**	2.64(0.18)	750,000	52.0

## Abbreviations

The following abbreviations are used in this manuscript:

GP	Gaussian process
GMM	Gaussian mixture models
DPMM	Dirichlet processes mixture model
PDF	Probability density function
CDF	Cumulative distribution function
SM	Spectral mixture kernel
SE	Squared exponential kernel
IM	Infinite mixture kernel

## Appendix A. Hierarchical Bayesian Models

A traditional Gaussian mixture model is expressed as:

(A1) $$p\left(y|\mu_{1},\ldots ,\mu_{k},s_{1},\ldots ,s_{k},\pi_{1},\ldots ,\pi_{k}\right)=\sum_{j=1}^{k}\pi_{j}N\left(\mu_{j},s_{j}^{-1}\right),$$

where $\mu_{j}$, $s_{j}$, and $\pi_{j}$ are the mean, the precision, and the proportional hyperparameters for the *j*-th components.

The mean $\mu_{j}$ is given Gaussain prior:

(A2) $$p\left(\mu_{j}|\lambda ,r\right)∼N\left(\lambda ,r^{-1}\right),$$

where λ and *r* are hyperparameters.

The hyperparameters λ and *r* are also given Gaussian and Gamma priors:

(A3) $$p\left(\lambda \right)∼N\left(\mu_{y},\sigma_{y}^{2}\right),$$

(A4) $$p\left(r\right)∼G\left(1,\sigma_{y}^{-2}\right)\propto r^{-1/2}\exp\left(-r\sigma_{y}^{2}/2\right),$$

where $\mu_{y}$ and $\sigma_{y}^{2}$ are the mean and variance of all observations.

Take Equation (A2) as the prior and Equation (A1) as the likelihood function, we can get the posterior distribution for the means $\mu_{j}$:

(A5) $$\begin{array}{cc} p(\mu_{j}|y_{j},s_{j},\lambda ,r)\; & \propto p\left(\mu_{j}|s_{j},\lambda ,r\right)p\left(y_{j}|\mu_{j},s_{j},\lambda ,r\right)\\ \; & \propto p\left(\mu_{j}|\lambda ,r\right)p\left(y_{j}|\mu_{j},s_{j}\right)\\ \; & \propto N\left(\mu_{j}|\lambda ,r^{-1}\right)\prod_{i=1}^{n_{j}}N\left(y_{j,i}|\mu_{j},s_{j}^{-1}\right)\\ \; & \propto \exp\left(-\frac{r}{2}{\left(\mu_{j}-\lambda \right)}^{2}-\frac{s_{j}}{2}\sum_{i=1}^{n_{j}}{\left(y_{j,i}-\mu_{j}\right)}^{2}\right)\\ \; & \propto \exp\left(-\frac{s_{j}n_{j}+r}{2}{\left(\mu_{j}-\frac{r\lambda +s_{j}n_{j}\bar{y_{j}}}{s_{j}n_{j}+r}\right)}^{2}\right)\\ \; & ∼N\left(\frac{r\lambda +s_{j}n_{j}\bar{y_{j}}}{s_{j}n_{j}+r},{\left(s_{j}n_{j}+r\right)}^{-1}\right), \end{array}$$

where $y_{j}$ is all observations belonging to class *j*, $n_{j}$ is the number of these observations, and ${\bar{y}}_{j}=\frac{1}{n_{j}}\sum y_{j,i}$.

Take Equation (A3) as the prior and Equation (A2) as the likelihood function, we get the posterior distribution for the hyperparameter λ:

(A6) $$\begin{array}{cc} p(\lambda |\mu_{1},\ldots ,\mu_{k},r)\; & \propto p\left(\lambda |r\right)p\left(\mu_{1},\ldots ,\mu_{k}|\lambda ,r\right)\\ \; & \propto p\left(\lambda \right)\prod_{j=i}^{k}p\left(\mu_{j}|\lambda ,r\right)\\ \; & \propto N\left(\lambda |\mu_{y},\sigma_{y}^{2}\right)\prod_{j=1}^{k}N\left(\mu_{j}|\lambda ,r^{-1}\right)\\ \; & \propto \exp\left(-\frac{1}{2\sigma_{y}^{2}}{\left(\lambda -\mu_{y}\right)}^{2}-\frac{r}{2}\sum_{j=1}^{k}{\left(\mu_{j}-\lambda \right)}^{2}\right)\\ \; & \propto \exp\left(-\frac{kr+\sigma_{y}^{-2}}{2}\left(\lambda -\frac{\mu_{y}\sigma_{y}^{-2}+r\sum_{j=1}^{k}\mu_{j}}{kr+\sigma_{y}^{-2}}\right)\right)\\ \; & ∼N\left(\frac{\mu_{y}\sigma_{y}^{-2}+r\sum_{j=1}^{k}\mu_{j}}{kr+\sigma_{y}^{-2}},{\left(kr+\sigma_{y}^{-2}\right)}^{-1}\right). \end{array}$$

Take Equation (A4) as the prior and Equation (A2) as the likelihood function, we get the posterior distribution for the hyperparameter *r*:

(A7) $$\begin{array}{cc} p(r|\mu_{1},\ldots ,\mu_{k},\lambda )\; & \propto p\left(r\right)\prod_{j}p\left(\mu_{j=1}^{k}|\lambda ,r\right)\\ \; & \propto G_{R}\left(r|1,\sigma_{y}^{-2}\right)\prod_{j=1}^{k}N\left(\mu_{j}|\lambda ,r^{-1}\right)\\ \; & \propto r^{-\frac{1}{2}}\exp\left(-\frac{\sigma_{y}^{2}}{2}r\right)\prod_{j=1}^{k}r^{\frac{1}{2}}\exp\left(-\frac{r}{2}{\left(\mu_{j}-\lambda \right)}^{2}\right)\\ \; & \propto r^{\frac{k-1}{2}}\exp\left(-\frac{1}{2}\left(\sigma_{y}^{2}+\sum_{j=1}^{k}{\left(\mu_{j}-\lambda \right)}^{2}\right)r\right)\\ \; & ∼G_{R}\left(k+1,\left(k+1\right){\left(\sigma_{y}^{2}+\sum_{j=1}^{k}{\left(\mu_{j}-\lambda \right)}^{2}\right)}^{-1}\right)\\ \; & ∼G\left(\frac{k+1}{2},2{\left(\sigma_{y}^{2}+\sum_{j=1}^{k}{\left(\mu_{j}-\lambda \right)}^{2}\right)}^{-1}\right). \end{array}$$

The precision of each component $s_{j}$ is given Gamma prior:

(A8) $$p\left(s_{j}|\beta ,w\right)∼G\left(\beta ,w^{-1}\right),$$

where β and *w* are hyperparameters with inverse Gamma and Gamma priors:

(A9) $$p\left(\beta^{-1}\right)∼G\left(1,1\right)⟹p\left(\beta \right)\propto \beta^{-3/2}\exp\left(-1/\left(2\beta \right)\right),$$

(A10) $$p\left(w\right)∼G\left(1,\sigma_{y}^{2}\right).$$

Take Equation (A8) as the prior and Equation (A1) as the likelihood function, we can get the posterior distribution for the means $s_{j}$:

(A11) $$\begin{array}{cc} p(s_{j}|y_{j},\mu_{j},\beta ,w)\; & \propto p\left(s_{j}|\mu_{j},\beta ,w\right)p\left(y_{j}|\mu_{j},s_{j},\beta ,w\right)\\ \; & \propto p\left(s_{j}|\beta ,w\right)p\left(y_{j}|\mu_{j},s_{j}\right)\\ \; & \propto G_{R}\left(s_{j}|\beta ,w^{-1}\right)\prod_{i=1}^{n_{j}}N\left(y_{j,i}|\mu_{j},s_{j}^{-1}\right)\\ \; & \propto s_{j}^{\frac{\beta }{2}-1}\exp\left(-\frac{\beta w}{2}w_{j}\right)\prod_{i=1}^{n_{j}}s_{j}^{\frac{1}{2}}\left(-\frac{s_{j}}{2}{\left(y_{j,i}-\mu_{j}\right)}^{2}\right)\\ \; & \propto s_{j}^{\frac{\beta }{2}-1+\frac{n_{j}}{2}}\exp\left(-\frac{w\beta }{2}s_{j}-\frac{1}{2}\sum_{i=1}^{n_{j}}{\left(y_{j,i}-\mu_{j}\right)}^{2}s_{j}\right)\\ \; & ∼G_{R}\left(\beta +n_{j},\left(\beta +n_{j}\right){\left(w\beta +\sum_{i=1}^{n_{j}}{\left(y_{j,i}-\mu_{j}\right)}^{2}\right)}^{-1}\right). \end{array}$$

Take Equation (A10) as the prior and Equation (A8) as the likelihood function, we get the posterior distribution for the hyperparameter *w*:

(A12) $$\begin{array}{cc} p(w|s_{1},\ldots ,s_{k},\beta )\; & \propto p\left(w|\beta \right)p\left(s_{1},\ldots ,s_{k}|w,\beta \right)\\ \; & \propto p\left(w\right)\prod_{j=1}^{k}p\left(s_{j}|w,\beta \right)\\ \; & \propto G_{R}\left(w|1,\sigma_{y}^{2}\right)\prod_{j=1}^{k}G_{R}\left(s_{j}|\beta ,w^{-1}\right)\\ \; & \propto w^{-\frac{1}{2}}\exp\left(-\frac{w}{2\sigma_{y}^{2}}\right)\prod_{j=1}^{k}w^{\frac{\beta }{2}}\exp\left(-\frac{\beta s_{j}}{2}w\right)\\ \; & \propto w^{\frac{k\beta -1}{2}}\exp\left(-\frac{1}{2}\left(\sigma_{y}^{-2}+\beta \sum_{j=1}^{k}s_{j}\right)w\right)\\ \; & ∼G_{R}\left(k\beta +1,\left(k\beta +1\right){\left(\sigma_{y}^{-2}+\beta \sum_{j=1}^{k}s_{j}\right)}^{-1}\right). \end{array}$$

Take Equation (A9) as the prior and Equation (A8) as the likelihood function, we get the posterior distribution for the hyperparameter β:

(A13) $$\begin{array}{cc} p(\beta |s_{1},\ldots ,s_{k},w)\; & \propto p\left(\beta |w\right)p\left(s_{1},\ldots ,s_{k}|\beta ,w\right)\\ \; & \propto p\left(\beta \right)\prod_{j=1}^{k}p\left(s_{j}|\beta ,w\right)\\ \; & \propto \beta^{-\frac{3}{2}}\exp\left(-\frac{1}{2\beta }\right)\prod_{j=1}^{k}G_{R}\left(s_{j}|\beta ,w^{-1}\right)\\ \; & \propto \beta^{-\frac{3}{2}}\exp\left(-\frac{1}{2\beta }\right)\prod_{j=1}^{k}\frac{s_{j}^{\frac{\beta }{2}-1}e^{-\frac{\beta ws_{j}}{2}}}{\Gamma \left(\frac{\beta }{2}\right){\left(\frac{2}{w\beta }\right)}^{\frac{\beta }{2}}}\\ \; & \propto \Gamma {\left(\frac{\beta }{2}\right)}^{-k}\beta^{-\frac{3}{2}}{\left(\frac{w\beta }{2}\right)}^{\frac{k\beta }{2}}\exp\left(-\frac{1}{2\beta }-\frac{\beta w}{2}\sum_{j}s_{j}\right)\prod_{j=1}^{k}s_{j}^{\frac{\beta }{2}-1}. \end{array}$$

The posterior distribution of β is in analytical form but not a stantard form. It can be proved that $p\left(\log\left(\beta \right)|s_{1},\cdots ,s_{k},w\right)$ is log-concave. Thus we can use adaptive rejection sampling method [32] to sample this distribution.

The proportional hyperparameters $\pi_{1},\ldots ,\pi_{k}$ are given a symmetric Dirichlet prior:

(A14) $$p\left(\pi_{1},\ldots ,\pi_{k}|\alpha \right)∼\mathrm{Dirichlet}\left(\alpha /k,\ldots ,\alpha /k\right)=\frac{\Gamma (\alpha )}{\Gamma {\left(\frac{\alpha }{k}\right)}^{k}}\prod_{j=1}^{k}\pi_{j}^{\frac{\alpha }{k}-1}.$$

The probability of all indicators $c_{j}$ under the condition where $\pi_{1},\ldots ,\pi_{k}$ are fixed is:

(A15) $$p\left(c_{1},\ldots ,c_{n}|\pi_{1},\ldots ,\pi_{k}\right)=\prod_{j=1}^{k}\pi_{j}^{n_{j}}.$$

We can integrate out the proportional hyperparameters and get the distribution of each indicator conditioning on α:

(A16) $$\begin{array}{cc} p\left(c_{1},\ldots ,c_{n}|\alpha \right)\; & =\int p\left(c_{1},\ldots ,c_{k}|\pi_{1},\ldots ,\pi_{k}\right)p\left(\pi_{1},\ldots ,\pi_{k}\right)d\pi \\ \; & =\int \frac{\Gamma (\alpha )}{\Gamma {\left(\frac{\alpha }{k}\right)}^{k}}\prod_{j=1}^{k}\pi_{j}^{\frac{\alpha }{k}-1}\prod_{j=1}^{k}\pi_{j}^{n_{j}}d\pi \\ \; & =\frac{\Gamma (\alpha )}{\Gamma {\left(\frac{\alpha }{k}\right)}^{k}}\int \prod_{j=1}^{k}\pi_{j}^{n_{j}+\frac{\alpha }{k}-1}d\pi \\ \; & =\frac{\Gamma (\alpha )}{\Gamma {\left(\frac{\alpha }{k}\right)}^{k}}\frac{\prod_{j=1}^{k}\Gamma \left(n_{j}+\frac{\alpha }{k}\right)}{\Gamma (n+\alpha )}\\ \; & =\frac{\Gamma (\alpha )}{\Gamma (n+\alpha )}\prod_{j=1}^{k}\frac{\Gamma \left(n_{j}+\frac{\alpha }{k}\right)}{\Gamma (\frac{\alpha }{k})}. \end{array}$$

When using Gibbs sampling methods to sample c, we have to sample each $c_{i}$ conditioning on others $c_{i}$. So we need to get the conditional distribution of $c_{i}$:

(A17) $$\begin{array}{cc} p(c_{i}=j|c_{-i},\alpha )\; & =\frac{p(c|\alpha )}{p(c_{-i}|\alpha )}\\ \; & =\frac{\frac{\Gamma (\alpha )}{\Gamma (n+\alpha )}\prod_{j=1}^{k}\frac{\Gamma \left(n_{j}+\frac{\alpha }{k}\right)}{\Gamma (\frac{\alpha }{k})}}{\frac{\Gamma (\alpha )}{\Gamma (n-1+\alpha )}\frac{\Gamma \left(n_{-i,j}+\frac{\alpha }{k}\right)}{\Gamma \left(n_{j}+\frac{\alpha }{k}\right)}\prod_{j=1}^{k}\frac{\Gamma \left(n_{j}+\frac{\alpha }{k}\right)}{\Gamma (\frac{\alpha }{k})}}\\ \; & =\frac{n_{-i,j}+\alpha /k}{n-1+\alpha }, \end{array}$$

where $n_{-i,j}$ is the number of the *j*-th components except the *i*-th point.

α is given an inverse gamma distribution:

(A18) $$p\left(\alpha^{-1}\right)∼G_{R}\left(1,1\right)⟹p\left(\alpha \right)\propto \alpha^{-\frac{3}{2}}\exp\left(-\frac{1}{2\alpha }\right).$$

Given α, the probability of the number of points for all components, $n_{1},\ldots ,n_{k}$ is:

(A19) $$p\left(n_{1},\ldots ,n_{k}|\alpha \right)=\frac{\alpha^{k}\Gamma \left(\alpha \right)}{\Gamma (n+\alpha )}.$$

Taking Equation (A18) as the prior and Equation (A19) as the likelihood function, we can get the posterior distribution of α:

(A20) $$\begin{array}{cc} p(\alpha |n_{1},\ldots ,n_{k})\; & \propto p\left(\alpha \right)p\left(n_{1},\ldots ,n_{k}|\alpha \right)\\ \; & \propto \alpha^{-\frac{3}{2}}\exp\left(-\frac{1}{2\alpha }\right)\frac{\alpha^{k}\Gamma \left(\alpha \right)}{\Gamma (n+\alpha )}. \end{array}$$
